# The effect of multiple single cannulation technique on complications of arteriovenous fistulae: A meta-analysis

**DOI:** 10.1097/MD.0000000000039748

**Published:** 2024-09-20

**Authors:** Peng Shu, Xia Wang, Zhuping Wen, Chenchen Li, Yiqi Luo, Fang Xu

**Affiliations:** a The Central Hospital of Wuhan, Tongji Medical College, Huazhong University of Science and Technology, China.

**Keywords:** area puncture, arteriovenous fistula, buttonhole cannulation, complication, multiple single cannulation technique, rope ladder cannulation

## Abstract

**Objective:**

To evaluate the effect of multiple single cannulation technique on the complications of arteriovenous fistula.

**Methods:**

A comprehensive literature search was conducted to investigate the impact of multiple single cannulation technique on the complications of arteriovenous fistula. The search was performed in both Chinese and English databases including Wanfang Medicine, China National Knowledge Infrastructure, Vip, Pubmed, Embase, and The Cochrane Library, with a search period up to December 20, 2023. Following literature screening and data extraction, the quality of the included studies was assessed using the Cochrane Bias Assessment Tool for Randomized Controlled Trials. Statistical analysis was performed using Review Manager version 5.3.

**Results:**

Thirteen papers, totaling 1299 patients, were included in the analysis. The experimental group consisted of 646 patients, while the control group had 595 patients. The meta-analysis revealed that the multiple single cannulation technique was more effective than rope ladder cannulation and buttonhole cannulation in reducing the incidence of angiomas (odds ratio [OR] = 0.19; 95% confidence interval [CI] = 0.10–0.35), stenosis (OR = 0.22; 95% CI 0.13–0.39), thrombosis (OR = 0.17; 95% CI = 0.07–0.39), and blood seepage (OR = 0.13; 95% CI = 0.08–0.21) of arteriovenous fistulas (*P* < .05). Additionally, it was found to increase the success rate of nurses’ single cannulation (OR = 4.20; 95% CI = 1.78–9.95) of arteriovenous fistulas (*P* < .05).

**Conclusion:**

Multiple single cannulation technique could effectively reduce the incidence of complications of arteriovenous fistula, improve the success rate of cannulation, prolong the life span of arteriovenous fistula, and prolong the survival cycle of hemodialysis patients.

## 1. Introduction

In recent years, there had been a gradual increase in the incidence of chronic kidney disease (CKD), which had a significant impact on the physical and mental health of the population. Epidemiological data indicate that the global prevalence of CKD was 13.4%, with the number of patients receiving renal replacement therapy ranging from 49.02 million to 70.83 million. In China, the prevalence of CKD among the population was10.8%, affecting approximately 120 million individuals.^[1]^ The 3 main treatment options for end-stage CKD were kidney transplant, hemodialysis, and peritoneal dialysis. However, due to factors such as the scarcity of kidney donors, the high cost of transplantation, the risk of infection associated with peritoneal dialysis, and the challenges in managing peritoneal dialysis patients, hemodialysis had become increasingly prevalent in clinical practice. Globally, approximately 69.4% to 91.9% of patients opt for hemodialysis, making it the most commonly chosen treatment for end-stage chronic kidney disease.^[2–4]^

According to the data from China’s hemodialysis case registration system, the number of hemodialysis patients in China reached 633,000 by the end of 2019.^[5]^ Hemodialysis access options mainly included arteriovenous fistula (AVF) and dialysis catheter with polyester. Approximately 77.12% to 88.20% of hemodialysis patients in China used arteriovenous fistula for dialysis.^[4,6]^ The Chinese hemodialysis vascular access consensus recommended that each dialysis center should use dialysis catheters for ≤10% of their patients, while the use of arteriovenous fistulas should be at least 80%.^[7]^ Hemodialysis patients typically underwent dialysis 2 to 3 times per week, which required cannulation of the arteriovenous fistula around 280 times per year. However, this frequent cannulation could have negative effects on the patient’s arteriovenous fistula. The most commonly used cannulation methods were rope ladder cannulation, buttonhole cannulation, and area puncture. Rope ladder cannulation required higher vascular conditions, with a minimum length of 6 cm and could cause complications such as hemangioma and arteriovenous fistula angiomatous dilatation, which in turn affect the patient’s cardiac function. Buttonhole cannulation also had higher implementation requirements, as it required the same nurse to use the same angle and depth for consecutive cannulations until a complete subcutaneous tunnel was established. However, buttonhole cannulation was prone to fistula infections, which could lead to bacterial toxemia and, in severe cases, patient death. It was important to note that guidelines and expert consensus did not recommend the use of area puncture.^[7,8]^

In recent years, a new cannulation method called multiple single cannulation technique has been proposed.^[9]^ Domestic and foreign research on multiple single cannulation technology was relatively limited. Portuguese scholars had demonstrated that this method could effectively reduce complications associated with arteriovenous fistula, such as hematoma, angioma, and stenosis.^[10,11]^ These complications could negatively impact dialysis adequacy and the lifespan of the arteriovenous fistula. Currently, there was a lack of uniformity in the cannulation methods used in different dialysis centers in China, and the results of various studies on arteriovenous fistula cannulation methods were inconsistent.^[12–16]^ Therefore, this paper aimed to compare the effects of multiple single cannulation techniques on arteriovenous fistula through a Meta-analysis using evidence-based medicine. The findings of this study would provide a basis for selecting the best cannulation method for nursing practice. The methodology and results of this study were presented below.

## 2. Methods

### 2.1. Inclusion exclusion criteria:

Inclusion criteria:

1. Dialysis patients with autologous arteriovenous fistulae.
2. Age ≥ 18 years.
3. Dialysis duration ≥ 3 months.
4. The type of study design was randomized controlled, with or without the use of blinding.
5. Interventions: multiple single cannulation techniques or fixed-point cannulation for patients in the experimental group and rope ladder cannulation or buttonhole cannulation for patients in the control group.
6. Outcome indicators: the rate of the thrombus, occlusion, hemangioma, verrucous dilatation, and hemorrhage, the success rate of a single cannulation, stenosis, and blood leakage.

Exclusion criteria: (1) unavailability of the original text, (2) effect sizes could not be converte,d (3) non-Chinese or non-English literature, (4) inability to extract complete data, and (5) duplicate publications, conference papers.

This systematic review and meta-analysis were conducted according to the guidelines of the Cochrane methodology and Preferred Reporting Items for Systematic Reviews and Meta-Analyses. This study was approved by the Ethics Committee of The Central Hospital of Wuhan, application number: WHZXKYL2023-155.This study had been registered on PROSPERO, Registration number was CRD42024497249.

### 2.2. Literature search

Searches were conducted in Wanfang Medical, China Knowledge Network, Vip, Pubmed, Embase, and The Cochrane Library databases using MeSH terms with free words. The search time limit was from the construction of the database to December 27, 2023. The date we conducted the literature search was January 3, 2024, and the date of data extraction was March 12, 2024.Take Pubmed for example: ((arteriovenous fistula cannulation[MeSH Terms) OR (rope ladder cannulation[MeSH Terms])) OR (buttonhole cannulation[MeSH Terms])) OR (area cannulation[MeSH Terms])) OR (multiple single cannulation[MeSH Terms])) AND (complications[MeSH Terms]))) OR (angiomas[MeSH Terms])) OR (thrombi[MeSH Terms])) OR (hematomas[MeSH Terms])) OR (hematomas[MeSH Terms])) OR (bleeding[MeSH Terms])) OR (cannulation success rate[MeSH Terms])) OR (randomized controlled cannulation[MeSH Terms]))) AND (randomized controlled[Title/Abstract]))).

### 2.3. Literature screening and data extraction

Literature was managed using Endnote X7, and 2 independent researchers (Luo Yiqi and Wen Zhuping) screened the literature and extracted data according to the literature inclusion and exclusion criteria for this study, with a third researcher (Wang Xia) exercising judgment when disagreements were encountered. The extracted data were cross-checked by 2 people and entered into Excel. The extracted data included the following: article title, author name, publication time, sample size of each group, study type, follow-up time, age, gender, dialysis age, follow-up time, and outcome indicators.

### 2.4. Evaluation of the quality of literature

Two authors independently evaluated the quality of the included studies using the Cochrane Risk of Bias 2 tool for randomised controlled trials.^[17]^ The tool involves assessing 7 domains: randomization process (selection bias), concealment of the allocation sequence (selection bias), blinding of participants and health professionals (performance bias), blinding of outcome assessment (detection bias), missing outcome data (attrition bias), selective reporting of results (reporting bias), and other potential sources of bias. Assessment decisions were categorized as “low risk of bias,” “high risk of bias,” or “some concerns” low risk, the quality of the literature was graded A. When some dimensions were evaluated as low risk and there was no high risk, the quality of the literature was graded B. When 1 dimension was evaluated as high risk, the quality of the literature was graded C.

### 2.5. Statistical methods

Meta-analysis of the literature was performed using Review Manager 5.3 statistical software. The heterogeneity of the literature was tested using the chi-square test, if *P* > .1 and I^2^ < 50%, indicating that there was no significant heterogeneity in the statistical results, a fixed-effects model was selected for analysis. If *P* < .1 and I^2^ > 50%, indicating that there was significant heterogeneity in the statistical results, the causes of heterogeneity were first analyzed, if there was no clinical heterogeneity, a random-effects model was selected for analysis; if clinical heterogeneity existed, a subgroup analysis was performed according to factors such as the type of complications. If there was no clinical heterogeneity, a random-effects model was chosen for analysis. Sensitivity analysis was used for publication to determine the stability of the studies, and funnel plots were used to determine the potential publication bias. The effect sizes of the outcome indicators were expressed as odds ratio (OR) with 95% confidence intervals (CIs), and *P* < .05 was used to indicate statistical significance.

## 3. Results

### 3.1. Results of Literature Screening

**Figure 1.**
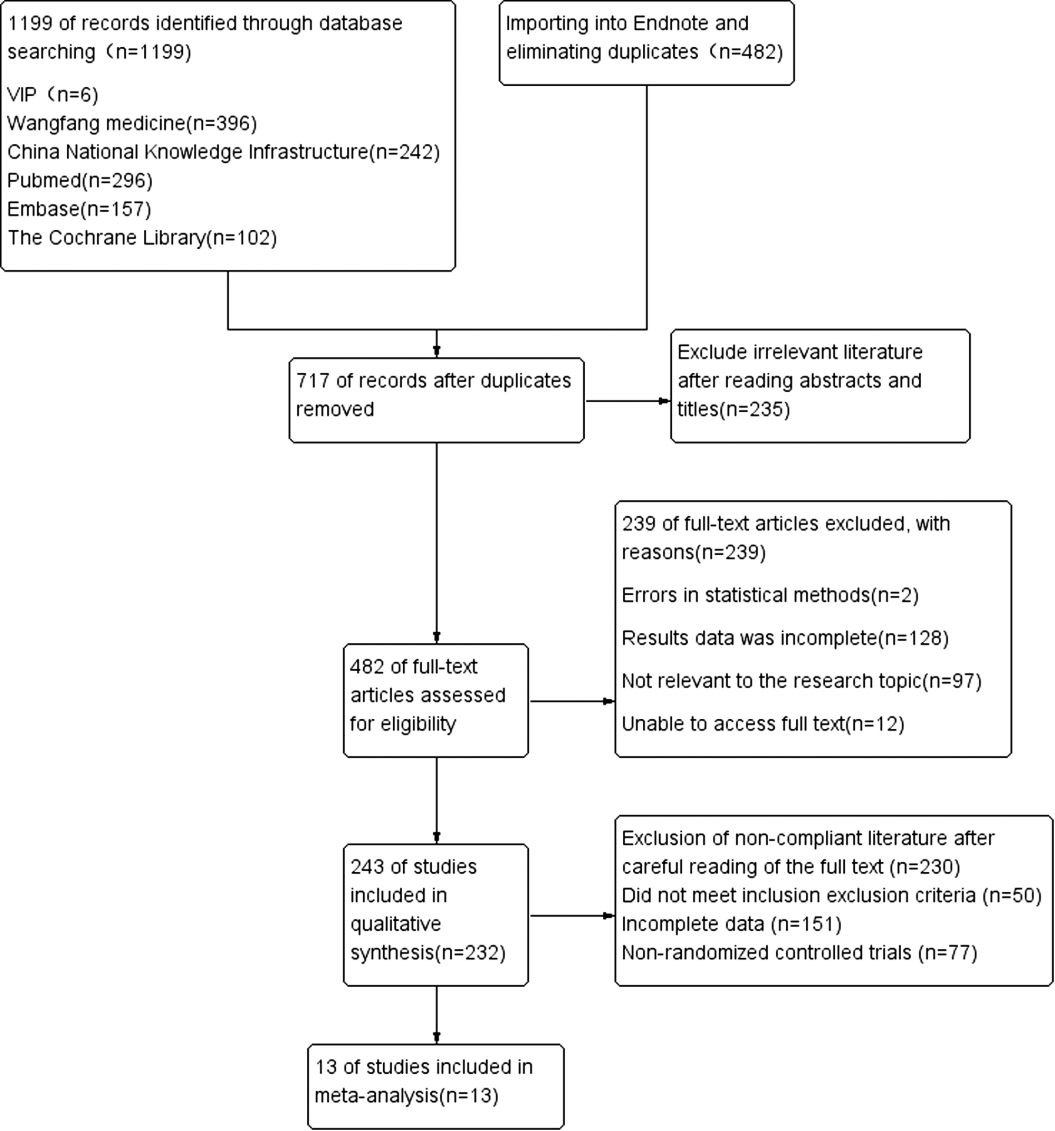
PRISMA flow diagram detailing the study selection process. PRISMA = Preferred Reporting Items for Systematic Reviews and Meta-Analyses.

The initial screening of the literature involved 1199 articles. Using Endnote software and manual screening, duplicates were removed, leaving 717 articles. After reading the abstract and title, 482 articles remained. Further reading of the full article reveals 243 articles relevant to our study. Finally, 13 articles were included in the study according to the inclusion and exclusion criteria, comprising 1 article in English^[11]^ and 12 articles in Chinese.^[16,18–28]^ The detailed Preferred Reporting Items for Systematic Reviews and Meta-Analyses flowchart of the study selection process was presented in Figure 1.

### 3.2. Basic characteristics of the included literature

**Figure 2.**
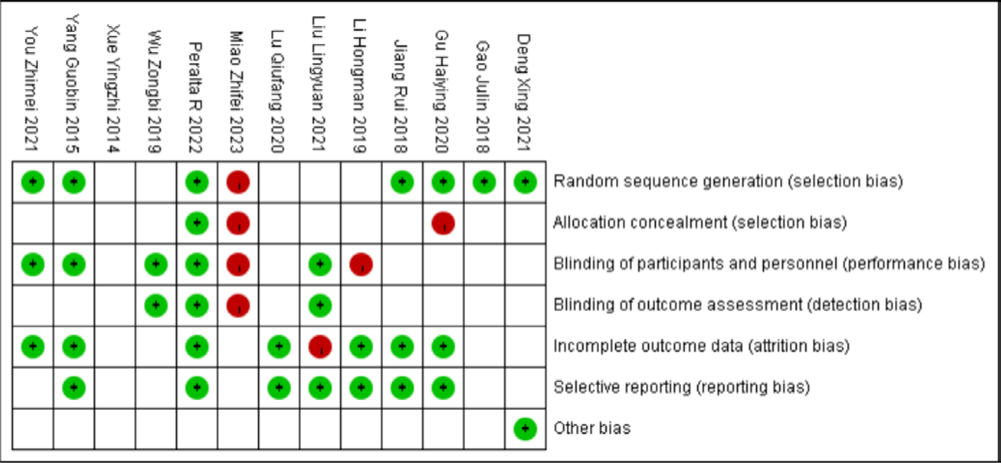
Literature publication bias risk map.

**Figure 3.**
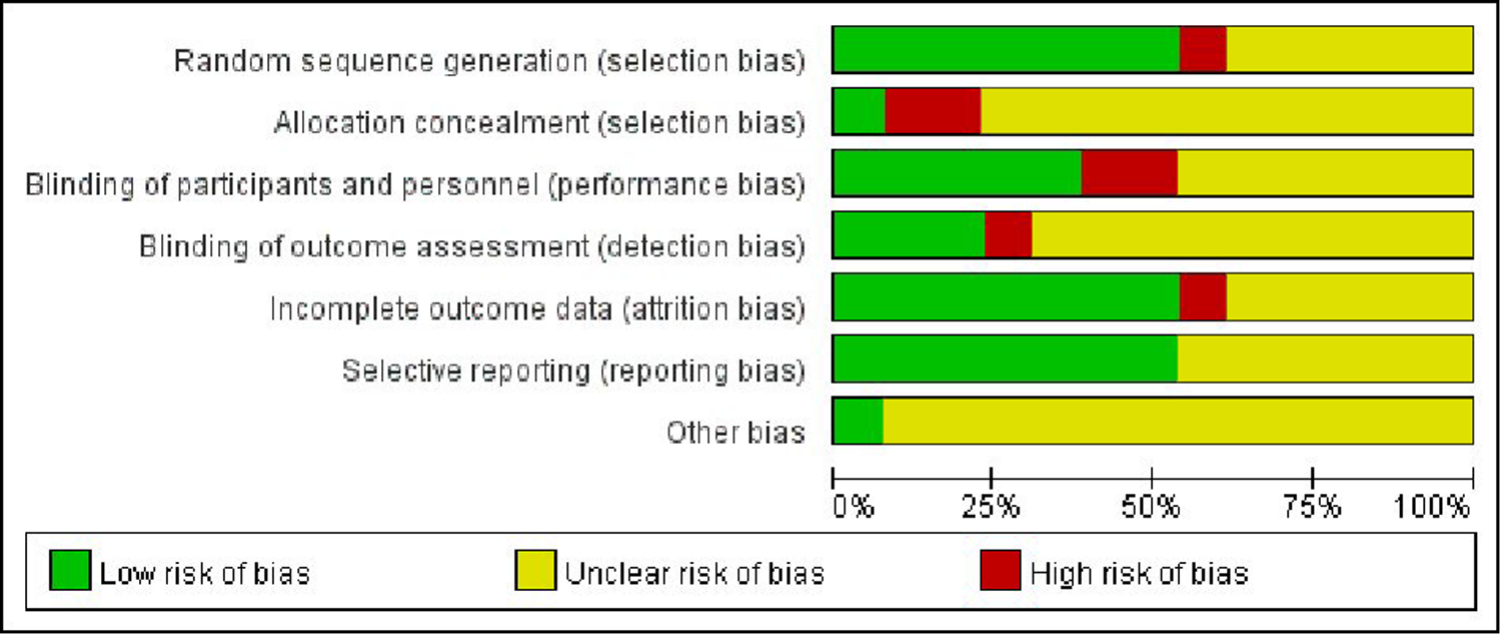
Literature publication bias graph.

This study included literature sources from 2 countries, spanning from 2014 to 2023. A total of 1299 hemodialysis patients who used autologous arteriovenous fistulae were examined. The study design consisted of randomized controlled trials, and the interventions tested were rope ladder cannulation, multiple single cannulation, and buttonhole cannulation. The experimental group comprised 646 cases, while the control group had 595 cases. The quality of the included literature was assessed using Figures 2 and 3. The characteristics of the included studies was presented in Table 1.

**Table 1:** Basic characteristics of the included literature

Year	Author	country	patients	Average age (yrs)	Sex (male)	Average age on dialysis (months)	AVF usage time (months)	Length of intervention (months)	outcomes
2021	You Zhimei^[18]^	China	50	58.14 ± 10.84/54.83 ± 15.10	30	43.77 ± 24. 46/74.05 ± 36.12	N	N	a,b,c,d
2015	Yang Guobin^[19]^	China	46	40.5 ± 4.9/44.1 ± 4.7	26	N	N	N	b,e,d,f
2014	Xue Yingzhi^[20]^	China	170	52.5 ± 14.5/50.85 ± 13.4	97	5.2 ± 3.2/4.9 ± 2.7	N	20	a,b,c,d,g
2019	Wu Zongbi^[21]^	China	43	50.76 ± 10.94/50.86 ± 7.74	31	N	N	6	a,b,f
2023	Miao Zhifei^[22]^	China	120	38.27 ± 8.39/40.55 ± 10.15	72	N	4.57 ± 2.98/3.98 ± 2.82	12	a,b,c,d, g
2020	Lu Qiufang^[16]^	China	84	45.38 ± 1.26/45.42 ± 1.37	50	N	N	12	a,b,f,g
2021	Liu Lingyuan^[23]^	China	72	63.0 ± 5.5/62.7 ± 5.8	39	N	N	9	a,b,c,d,f
2019	Li Hongman^[24]^	China	97	49.09 ± 13.66/50.52 ± 13.59	42	7.66 ± 4.71/8.02 ± 4.96	N	8	b,f,g
2018	Jiang Rui^[25]^	China	60	N	28	N	N	9	b,c,d,g
2020	Gu Haiying^[26]^	China	89	47.0 ± 11.7/47.3 ± 12.0	47	N	18.04 ± 11.50/17.51 ± 11.90	12	c,b,f,g
2018	Gao Julin^[27]^	China	128	45.69 ± 14.58/45.95 ± 14.65	75	14.16 ± 13.80/15.00 ± 14.04	N	6	a,b,c,g
2021	Deng Xing^[28]^	China	168	N	98	N	N	12	a,f,g
2022	Peralta, R^[11]^	Portugal	172	68.46 (57–80)/68.74 (56–79.50)	134	22.84 (2–10)/15.31 (2–8)	8.79 (1.9–6.30)/11.27 (2.10–8.30)	12	b,d,e

Note: a: stenosis, b: hemangioma c: success rate of 1 cannulation, d: thrombus,e: insufficient distal perfusion,f: occlusion of fistula,g: blood leakage, N: denotes failure to extract data in the literature.

AVF = arteriovenous fistula.

### 3.3. Meta-analysis results

#### 3.3.1. Effect of multiple single cannulation technique on the incidence of angiomas in arteriovenous fistulae

**Figure 4.**
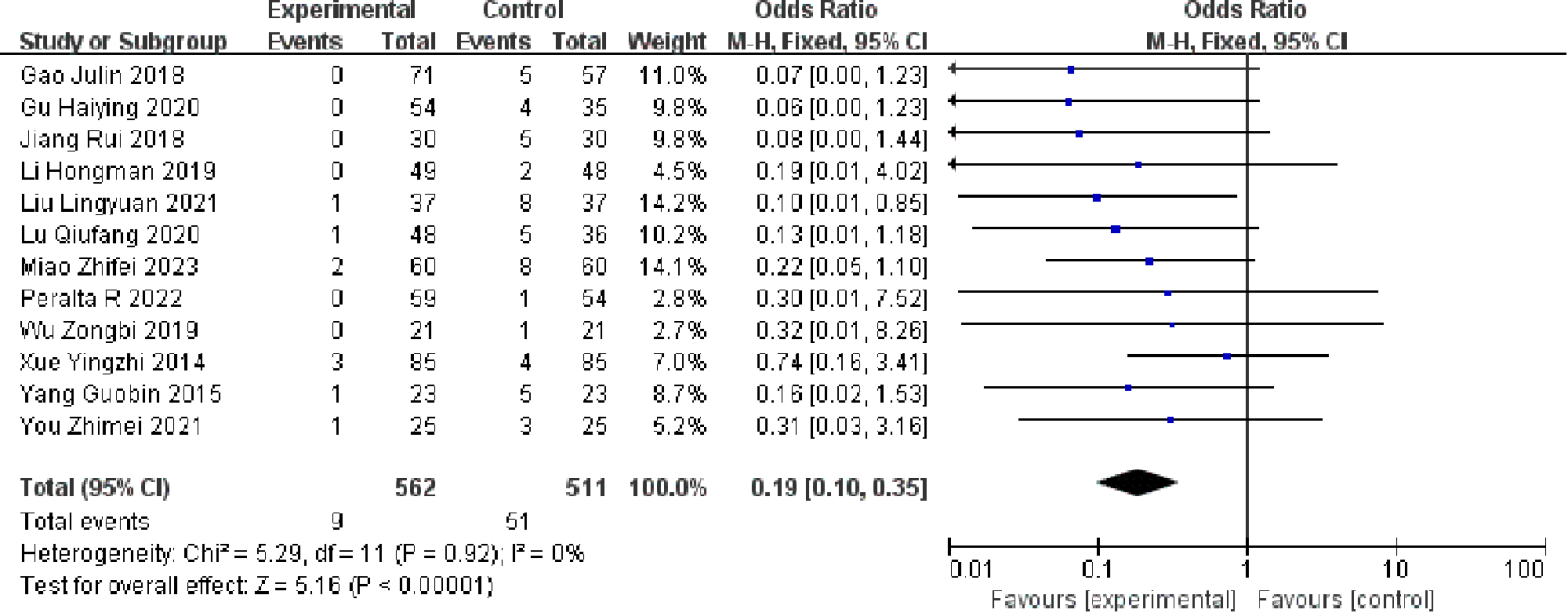
Forest plot of the effect of multiple single cannulations technique on the incidence of angiomas.

**Figure 5.**
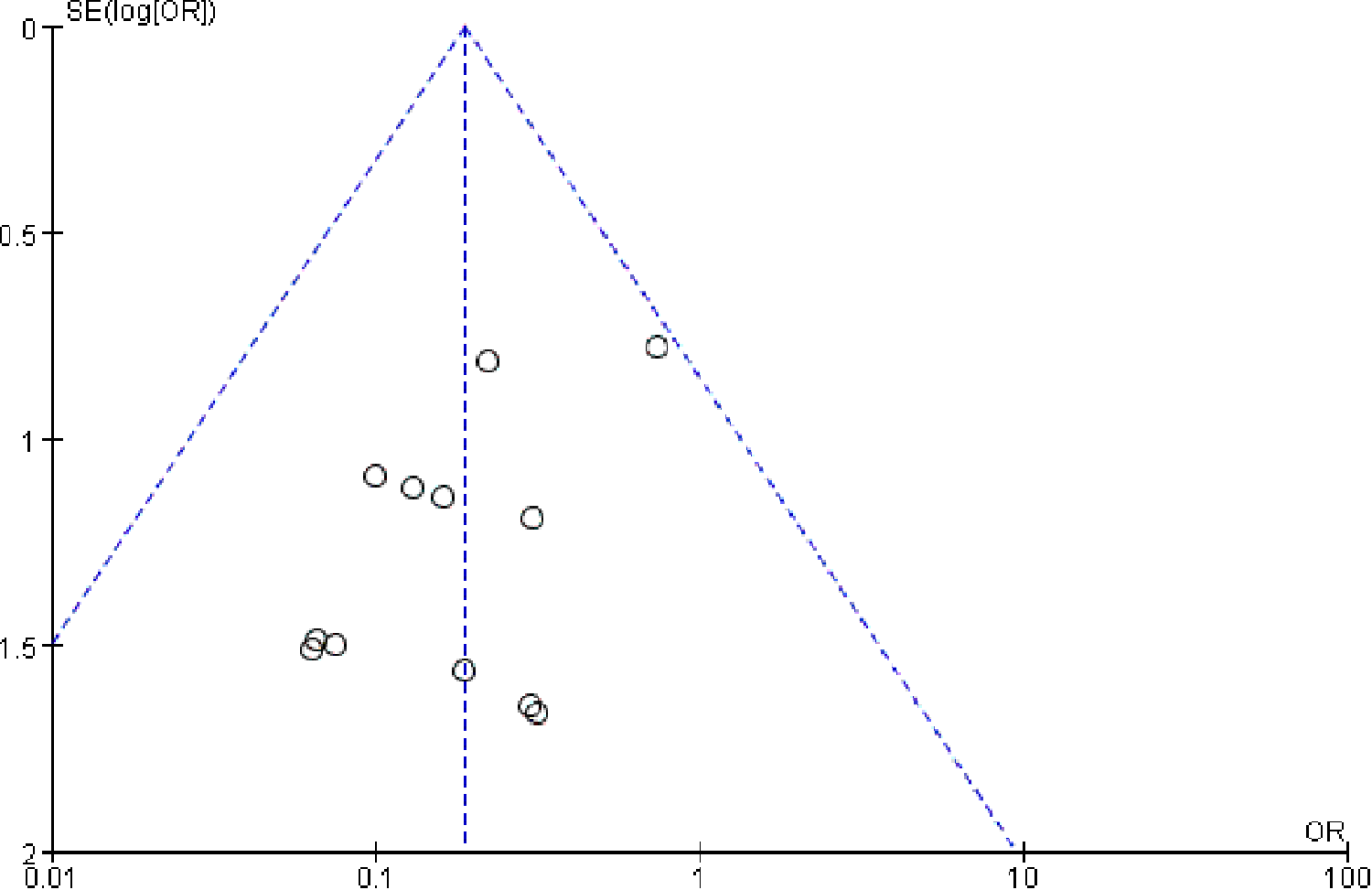
Funnel plot of the effect of multiple single cannulation technique on the incidence of hemangiomas.

The effect of multiple single cannulation technique on the incidence of angiomas in arteriovenous fistulae was evaluated in twelve papers involving hemodialysis patients. The results, which showed no heterogeneity among the included literature (I^2^ = 0%, *P* = .92), indicated that the multiple-single cannulation technique was effective in reducing the incidence of aneurysms when compared to rope ladder cannulation and buttonhole cannulation (OR = 0.19, 95% CI =  0.10–0.35, *P* < .05). This difference was statistically significant (Fig. 4). The combined results of the literature were subjected to a funnel plot test, which revealed no significant asymmetry in the scatter points,indicating minimal publication bias as shown in Figure 5.

#### 3.3.2. Effect of multiple single cannulation techniques on the incidence of arteriovenous fistulae stenosis

**Figure 6.**
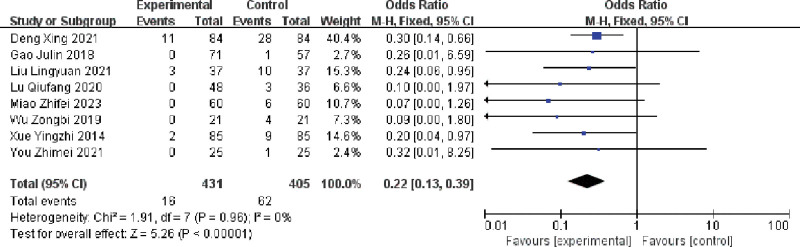
Forest plot of the effect of multiple single cannulation technique on stenosis.

A total of 8 publications assessing the effect of multiple single cannulation techniques on the incidence of AVF stenosis in patients with arteriovenous fistula.^[16,18,20–23,27,28]^ The results showed (I^2^ = 0%, *P* = .96), there was no heterogeneity in the included literature, and using a fixed-effects model, multiple single cannulation was able to reduce the incidence of AVF stenosis compared with rope ladder cannulation and buttonhole cannulation (OR = 0.22, 95% CI = 0.13–0.39, *P* < .05], and the difference was statistically significant, as shown in Figure 6.

#### 3.3.3. Effect of multiple single cannulation techniques on the incidence of thrombosis in arteriovenous fistulae

6 publications assessing the impact of multiple single cannulation technique on the incidence of thrombosis in arteriovenous fistulae.^[11,19,20,22,23,25]^

**Figure 7.**
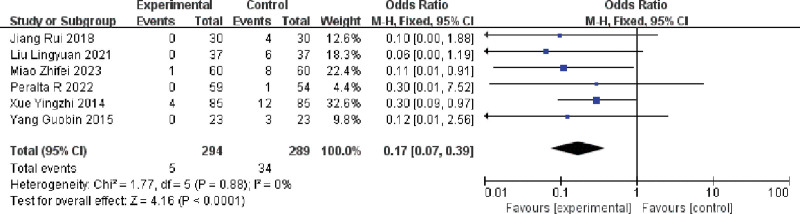
Forest plot of the effect of multiple single cannulation technique on the incidence of thrombosis.

The results showed (I^2^ = 0%, *P* = .88),there was no heterogeneity in the included literature, and using a fixed-effects model, multiple single cannulation techniques reduced the incidence of arteriovenous fistula thrombosis compared with rope ladder cannulation and buttonhole cannulation [OR = 0.17, 95% CI = 0.07–0.39, *P* < .05], and the difference was statistically significant, as shown in Figure 7.

#### 3.3.4. Effect of multiple single cannulation techniques on the incidence of arteriovenous fistula

**Figure 8.**
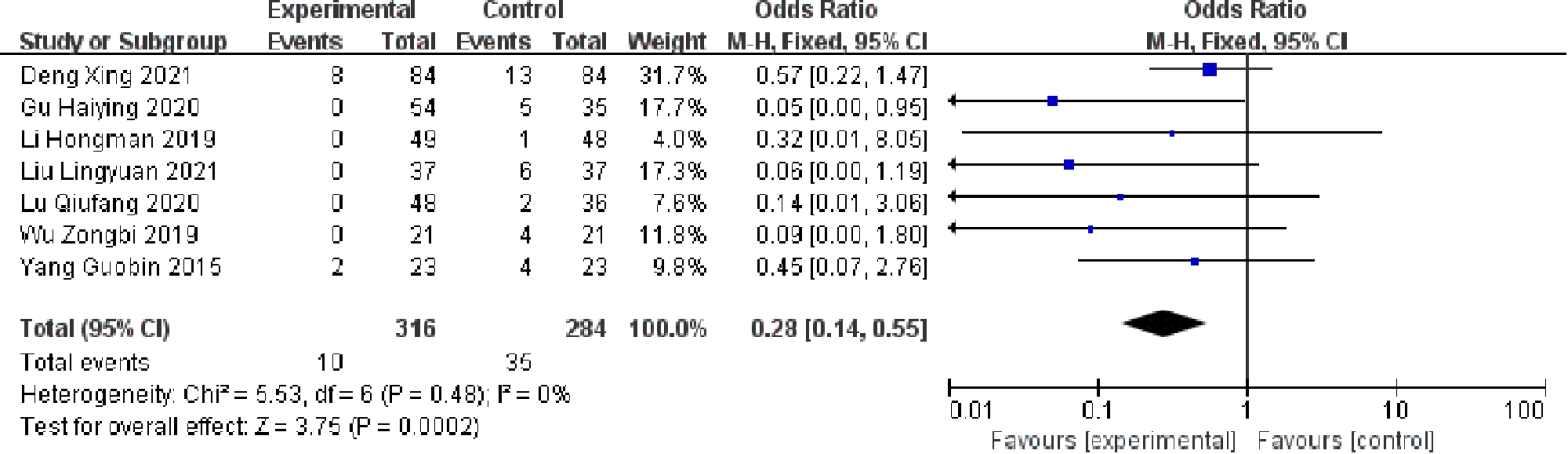
Forest plot of the effect of multiple single cannulation technique on the incidence of internal fistula occlusion.

Seven publications assessed the effect of multiple single cannulation techniques on the incidence of AVF occlusion in patients with arteriovenous fistulae.,^[16,19,21,23,24,26,28]^ the results showed (I^2^ = 0%, *P* = .48),there was no heterogeneity in the included literature, and using a fixed-effects model, multiple single cannulation reduced the incidence of arteriovenous fistula occlusion compared with rope ladder cannulation and buttonhole cannulation [OR = 0.28, 95% CI = 0.14–0.55, *P* < .05], and the difference was statistically significant, as shown in Figure 8.

#### 3.3.5. The effect of multiple single cannulation techniques on the incidence of blood leakage from arteriovenous fistulae

**Figure 9.**
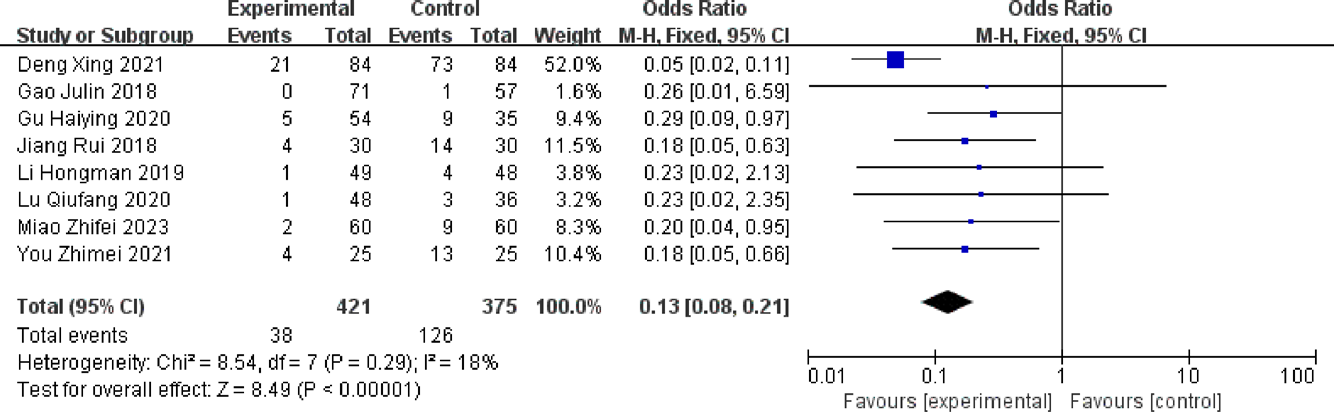
Forest plot of the effect of multiple single cannulation technique on the incidence of blood seepage.

Eight publications assessing the effect of multiple single cannulation techniques on the incidence of blood leakage in patients with arteriovenous fistulae.^[16,18,22,24–28]^ The results showed (I^2^ = 18%, *P* = .29),there was mild heterogeneity in the included literature, and using a fixed-effects model, multiple single cannulation was able to reduce the incidence of blood oozing from the arteriovenous fistula compared to rope ladder cannulation and buttonhole cannulation (OR = 0.13, 95% CI = 0.08–0.21, *P* < .05), the difference was statistically significant, as shown in Figure 9.

#### 3.3.6. Effect of multiple single cannulation techniques on the success rate of a single cannulation of arteriovenous fistula

**Figure 10.**
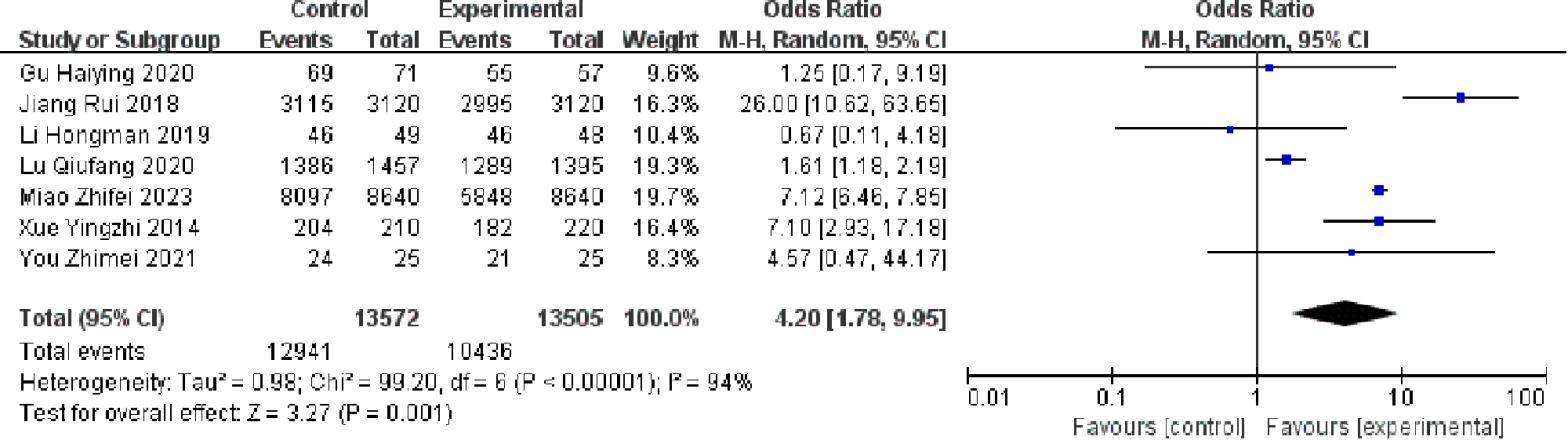
Forest plot of the effect of multiple single cannulation on the success rate of a single cannulation.

Seven publications evaluating the effect of multiple single cannulation techniques on the success rate of primary cannulation in patients with arteriovenous fistulae.^[16,18,20,22,24–26]^ The results showed (I^2^ = 94%, *P* < .1) there was heterogeneity in the included literature and analyzed using a random-effects model, multiple single cannulations were able to improve the success rate of primary cannulation of arteriovenous fistula compared with cord cannulation and buttonhole cannulation (OR = 4.20; 95% CI = 1.78–9.95, *P* < .05), and the difference was statistically significant, as shown in Figure 10.

## 4. Discussion

### 4.1. Effect of multiple single cannulation techniques on the incidence of arteriovenous fistula angiomas

When arteriovenous fistula blood vessels developed aneurysmal dilatation, it resulted in thinning of the skin epidermis. Continuous cannulation could easily lead to rupture and hemorrhage of the angiomas, posing a life-threatening risk to the patient. The occurrence of arteriovenous fistula angiomas varied from 5% to 60% in foreign countries, while a cross-sectional study involving 2547 patients in Hainan Province revealed a prevalence of 41.22% for AVF angiomas.^[29]^ The primary reasons for the development of arteriovenous fistula angiomas in patients were as follows: (1) repeated cannulation causing damage to smooth muscle cells during dialysis, leading to intimal hyperplasia; (2) narrowing of the fistula, resulting in increased pressure in the anterior segment and consequent dilation of the blood vessel lumen; (3) end-stage renal disease causing inflammatory factors, toxins, and other damage to the blood vessels; (4) increased venous blood flow speed due to surgery, leading to heightened internal vascular shear forces; and (5) genetic factors. The study demonstrated that the incidence of intra-arterial fistula hemangiomas was 1.73 times higher with area puncture compared to rope ladder cannulation, and 4.27 times higher compared to buttonhole cannulation.^[29]^ Meta-analysis of this study showed that multiple single cannulation technique were able to reduce the incidence of intra-arterial fistula hemangiomas compared to rope ladder and buttonhole cannulation, which might be attributed to the fact that multiple single cannulation techniques had fewer cannulation sites, which resulted in less injury to the smooth muscle as well as reducing the risk of hemangiomas formed by intraventricular blood impingement on the cannulation site.^[30]^ A meta-analysis showed that buttonhole cannulation was effective in reducing the incidence of hemangiomas.^[31]^ However, buttonhole cannulation was difficult to perform in clinical practice, making it more difficult to form and increasing the rate of infection in patients.^[32]^ However, multisingle cannulation techniques did not require the formation of subcutaneous tunnels, the operation was simple, and could be carried out better in clinical practice, but also not because of the lack of subcutaneous tunnels, thus reducing the chances of arteriovenous fistula infection.

### 4.2. Effect of multiple single cannulation techniques on the incidence of stenosis and thrombosis in arteriovenous fistulae

Recent studies had shown that mature arteriovenous fistulae will gradually lose function with the increase of dialysis time, and the patency rate at 2 years was 75%, and stenosis was one of the main reasons for the loss of function of arteriovenous fistulae.^[33]^ The mechanism of arteriovenous fistula stenosis was: (1) in the process of arteriovenous fistula maturation, the vessel wall of the vein will be thickened, the lumen will be dilated; (2) dialysis constant cannulation leads to venous blood vessel wall endothelial thickening, the formation of nonthrombotic stenosis; (3) inflammatory factors in the body, the toxin constant stimulation of the blood vessel wall formation of stenosis; (4) thrombus leads to arteriovenous fistula vessel stenosis.^[34]^ The most common caused of thrombosis of the arteriovenous fistula was the stenosis of the arteriovenous fistula due to the thickening of the intima-media wall of the venous vessels.^[35]^ Tordoir’s study showed that 85% of arteriovenous fistula thrombosis was caused by arteriovenous fistula stenosis.^[36]^ Therefore, the occurrence of stenosis and thrombosis in arteriovenous fistulas were mutually reinforcing. In our study, we showed that multiple single cannulation techniques was more effective in reducing the incidence of arteriovenous fistula stenosis and thrombosis than rope ladder cannulation and buttonhole cannulation. Meta-analysis by Wang Liping et al^[37]^ showed that buttonhole cannulation was more effective in reducing the incidence of arteriovenous fistula stenosis than rope ladder cannulation, which indirectly supports our study. Indirectly, this supported our findings. The reason for this might be that multiple single cannulation techniques was less damaging to the arteriovenous fistula vessels due to fewer cannulation points, and at the same time, changing the cannulation points reduces the possibility of endothelial hyperplasia in the arteriovenous fistula vessel wall. Some studies had shown that there would be granulation tissue around the tunnel of the button, and there would be skin hyperplasia in the vicinity of the granulation tissue, but the hyperplasia will subside after 3 months of changing the cannulation points.^[38]^ Multiple single cannulation technique sited with fewer cannulation sites and different cannulation sites for each dialysis session reduced the chance of vascular tissue hyperplasia in the arteriovenous fistula, thus reducing the likelihood of thrombosis and stenosis in the arteriovenous fistula.

### 4.3. Effect of multiple single cannulation techniques on the incidence of blood leakage from arteriovenous fistulae

There was a lack of definition and quantification of blood leakage from an arteriovenous fistula.^[39]^ The occurrence of blood leakage was related to the size of the needle used during cannulation, the nurse’s cannulation technique, and the use of anticoagulants and subcutaneous fat.^[40]^ In our study, we showed that multiple single cannulations were more effective than rope ladder cannulation and buttonhole cannulations in reducing blood seepage from arteriovenous fistulas, probably because multiple single cannulation techniques with repeated cannulation at fixed cannulation points resulted in endothelial hyperplasia in the patient’s arteriovenous fistula, which altered the physiologic conditions of the vessels and changed their brittleness, thus decreasing the incidence of blood seepage.

### 4.4. Effect of multiple single cannulation technique on the success rate of a single cannulation

The vascular condition of the patient was closely related to the patient’s underlying disease.^[35]^ However, the cannulation success rate was related to the nurse’s cannulation technique as well as the patient’s vascular condition.^[40]^ Fistula surgery results in a change in the anatomy of the patient’s veins, making it more difficult for nurses to cannulation the vessels. The current study suggested that buttonhole cannulation improves the success rate of single cannulation of arteriovenous fistula.^[41]^ The results of our study showed that multiple single cannulation technique had a higher success rate of single cannulation than rope ladder cannulation and buttonhole cannulation, and the results of meta-analysis by Yang et al^[30]^ also showed that buttonhole blunt-needle cannulation was able to increase the success rate of 1 cannulation in arteriovenous fistula compared with rope ladder cannulation, which was similar to our results. The reason for this might be that multiple single cannulation techniques caused endothelial hyperplasia of the venous wall of the arteriovenous fistula, resulting in a thickening of the vessel wall, which changed the brittleness of the vessel, thus increasing the success rate of the cannulation.

## 5. Conclusion

Through a meta-analysis of 13 randomized controlled studies, this study concluded that multiple single cannulation technique can effectively reduce the incidence of angiomas, thrombosis, blood leakage, and stenosis in arteriovenous fistulae. Additionally, these techniques increase the success rate of nurses’ single cannulation, reduce complications associated with arteriovenous fistulae, and prolong the use of arteriovenous fistulae. Ultimately, this leads to an extended life cycle for hemodialysis patients.

## 6. Limitations

The researcher is uncertain if some of the included literature in the study was blinded to patients, which could have affected the results. There is a lack of similar literature from foreign studies, which has the potential to influence the population use of multiple single cannulation technique. The definition of multiple single cannulation technique varies both nationally and internationally, so there may be relevant literature not yet included in the study that could impact the results.

## Acknowledgments

The researcher is uncertain if some of the included literature in the study was blinded to patients, which could have affected the results. There is a lack of similar literature from foreign studies, which has the potential to influence the population’s use of multiple single cannulation technique. The definition of multiple single cannulation technique varies both nationally and internationally, so there may be relevant literature not yet included in the study that could impact the results.

## Author contributions

**Writing—original draft:** Peng Shu.

**Data curation:** Xia Wang, Zhuping Wen, Chenchen Li, Yiqi Luo.

**Funding acquisition:** Fang Xu.

**Writing—review and editing:** Fang Xu.
